# Active Involvement of End-Users in an EHR Procurement Process: a Usability Walkthrough Feasibility Case Study

**DOI:** 10.1007/s11606-023-08277-2

**Published:** 2023-10-05

**Authors:** Romaric Marcilly, Blake Lesselroth, Sandra Guerlinger, Annick Pigot, Jessica Schiro, Sylvia Pelayo

**Affiliations:** 1 Univ. Lille, CHU Lille, ULR 2694 - METRICS: Évaluation des technologies de santé et des pratiques médicales, Lille, F-59000 France; 2 Inserm, CIC-IT 1403, F-59000 Lille, France; 3 School of Health Information Science, University of Victoria, Victoria, Canada; 4 University of Oklahoma-Tulsa, School of Community Medicine, Tulsa, USA; 5 CHU de Lille, Direction des Ressources Numériques, Lille, France; 6 Lille Catholic Hospitals, Information Department, Lille, F-59160 France

## INTRODUCTION

### Background

Government usability standards—including those from the US Office of the National Coordinator (ONC) of Health Information Technology (HIT)—emphasize the importance of user-centered design to maximize electronic health record (EHR) usability.^[1]^ Published studies^[2,3]^ and best-practice recommendations^[4,5]^ show that improving user experience can promote technology adoption, increase system efficiency^[6,7]^, improve patient safety^[8–10]^, and reduce clinician burnout.^[11]^ Nevertheless, clinicians cite EHR design as one of the most common causes of clinical errors, long work hours, and dissatisfaction.^[12,13]^ Furthermore, not all vendors—including those headquartered in the US—integrate user-centered design principles into the product lifecycle.^[2,14]^ It is incumbent upon healthcare organizational leaders and clinical champions to be actively engaged throughout implementation from technology procurement, through system configuration, to eventual deployment and training.

How a clinician uses an EHR depends on the system's work context and socio-technical characteristics (i.e., technology, organizational climate, and user).^[15–17]^ The specialty and practice settings are critically important to consider. For example, an emergency physician will not use the EHR like a geriatrician—each will have specific needs within their workflow. It is, therefore, a best practice to include end-users in the EHR procurement process.^[18–21]^

The methods organizational leaders use to engage end-users can influence the selection process outcome.^[22–25]^ While executives often only invite end-users to EHR demonstrations,^[24]^ there are practical ways for users to evaluate products during demonstrations. Project managers can administer the Usability Questionnaire for Demonstrations in Procurement (DPUQ)^[26]^ to gather end-user perceptions. Usability experts can then supplement this with a heuristic evaluation during the demonstration (HED).^[27]^ The HED is a low-cost method where an expert rates a product against a set of usability heuristics.^[28–30]^

These methods notwithstanding, data gathered during vendor demonstrations may not be the best quality since users rarely interact with the EHR, and sales representatives can hide product weaknesses.^[31]^ Prospective customers can gather more predictive data by conducting usability tests with clinical information processing scenarios (CLIPS). CLIPS are scripts representing clinical situations with tasks for users to complete while experts observe and collect data.^[23,31,32]^ Stakeholders, however, may be apprehensive about the time and cost of simulation testing of multiple EHRs with CLIPS.^[33,34]^ Heuristic evaluations are typically more cost-effective but do not involve end-users and can miss important issues.^[22,23,28]^ We see a need to combine these methods into a single protocol.

### Problem Statement and Research Questions

Schumacher and colleagues described a pragmatic approach to involve end-users in the procurement process: the usability walkthrough.^[34]^ During a usability walkthrough, end-users complete CLIPS with the EHR and classify usability problems using a set of heuristics. After the walkthrough, participants rate effectiveness, efficiency, and satisfaction.

A usability walkthrough is like simulation testing in that it involves end-users. In user simulation testing, experts identify problems while watching users test the technology, whereas, in a usability walkthrough, users identify the problems.^[28,34,35]^ The usability walkthrough is also like a cognitive walkthrough—both methods require users to think and talk through a clinical scenario. However, the cognitive walkthrough evaluates product learnability by asking standardized questions about interface intuitiveness and the ability to guide users through tasks.^[35,36]^ The usability walkthrough, by contrast, measures multiple usability dimensions by replacing standardized questions with heuristics. To conduct a usability walkthrough correctly, usability professionals train the end-users to apply the same heuristics experts use.

To summarize, usability walkthroughs permit users to compare EHRs without vendor interference and allow clinicians to champion their needs and preferences.^[34]^ Yet, no studies have formally investigated the feasibility of conducting a usability walkthrough during an EHR procurement. We sought to close this knowledge gap by asking four questions: (1) Are end-users able to detect, describe, and prioritize usability problems? (2) Does the usability walkthrough method help identify problems only detectable by clinical experts? (3) How satisfied are end-users with the usability walkthrough process? (4) What are the challenges of implementing a usability walkthrough during EHR procurement? To answer these questions, we conducted an implementation study of a usability walkthrough as a hospital transitioned to a new commercial EHR. In this article, we report on the method's feasibility. The results of the EHR evaluation are published elsewhere.^[37]^

## METHODS

### Study Context

Leadership at a private, non-profit, 1000-bed teaching hospital in Lille, France, issued a request for proposals (RFP) and organized a procurement process to select a replacement for their current commercial EHR. The process included three steps: (1) a vendor demonstration; (2) a usability walkthrough with each candidate EHR; and (3) technical and economic comparisons of EHRs selected during the walkthrough. The comparisons focused on “back-end” functionality (e.g., data interoperability). In this manuscript, we describe the second step of the process (i.e., usability walkthrough).

To conserve resources and adhere to a timeline, it was necessary to quickly thin the pool of EHR candidates for later technical evaluation. We did not set out to exhaustively safety test products or generate summative statistics during the second phase. Therefore, we limited the number of users recruited.

### Usability Workshop Implementation

**Figure 1:**
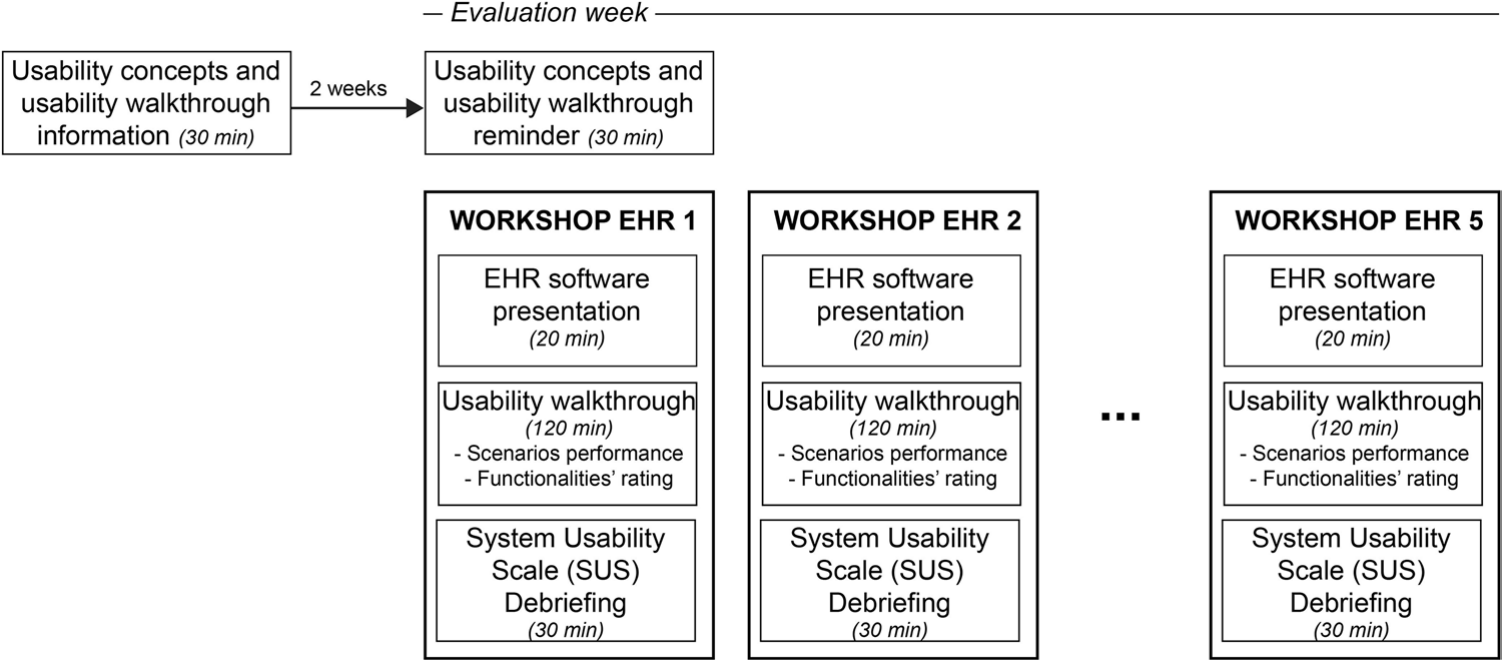
Evaluation process.

The project manager (AP) and four usability experts (RM, SG, JS, SP) designed a usability instructional session and a usability workshop that included structured exercises and evaluation instruments. We planned to conduct two instructional sessions and five workshops over three weeks from September to October 2020 (Fig. 1).

#### Usability Instructional Sessions

We hosted two face-to-face instructional sessions. We held the first session two weeks before the workshops and a refresher on the day of the first workshop. In both sessions, we explained the dimensions of usability, demonstrated the walkthrough method, reviewed usability assessment criteria (adapted from Scapin and Bastien)^[29]^, and introduced a usability issue severity rating scale. We answered questions and furnished the participants with a written summary of all content.

#### Workshop Design

##### **Preparation and Participants**

We received proposals from five vendors and scheduled five 3-hour workshops over one week: one per candidate (Fig. 1). During each workshop, the vendor presented their EHR to stakeholders. We then excused vendors from the proceedings; they were not permitted to interact with end-users during any other evaluation stage—including the usability walkthrough and end-user debriefing session (outlined below).

A multidisciplinary team of clinical representatives, the procurement manager, and a usability expert (JS) identified common inpatient EHR use scenarios and concerned end-users. We designed seven CLIPS simulating 59 EHR tasks (Appendix A), targeting frequently used or critical functionalities (Table 1). We also created a clinical dataset for the EHR. We sent the CLIPS and dataset to the EHR vendors two weeks before the usability sessions.

**Table 1:** List of End-User Profiles and Summary of End-User Tasks During Clinical Information Processing Scenarios (CLIPS)

Profile	Abstract of CLIPS and tasks
Pharmacist (*n* = 1)	Validation of a prescription, entry of medication dispensation
Admissions officer (*n* = 1)	Entry of new patient’s administrative data, of administrative discharge, of consultation and day hospital pre-admissions
Secretary (*n* = 1)	Dictation transcription, search and entry of appointments bookings for consultation and hospitalization
Nurses (*n* = 3)	Patient's arrival in the emergency room: entry of reason for admission, vital parameters, patient prioritization, medication administration, patient orientation, transfer to another service Patient hospitalization: entry of the bed installation, of the entry synthesis, of the initial clinical synthesis, of the autonomy assessment, of the Braden score, of the blood test, of the care planning, of the therapeutic administration, of the care delivery, of the traceability of a bandage, of the urinary catheter insertion, of the department exit, visualization of the care plan, correction of an error, update of a targeted transmission
Physicians (*n* =3)	Patient's arrival in the emergency room: entry of medical observation, prescription, medical decision, and coding Patient hospitalization: entry of medical history and disease, of the usual treatment, of allergies, of the initial prescription, of the progress note, of the discharge order and coding, visualization of laboratory results, of the care plan, of the prescriptions, of the medical observations and of the vital parameters, change of prescription, request for infectious advice, dictation of discharge letter.

We recruited nine end-users: three physicians (an emergency physician from the emergency unit, a cardiologist from the cardiology unit, and a neurologist from the geriatrics unit), three nurses (from emergency, cardiology, and geriatrics units), one pharmacist (from the central pharmacy), one medical clerk, and one admission officer (i.e., non-clinical staff member trained to manage administrative and logistic duties). Participants volunteered for the workshops; they were not compensated for their participation. None had been trained to use the candidate EHRs.

##### **Usability Walkthrough**

We organized participants into four evaluation groups—each supervised by a usability expert. Each group completed CLIPS at a computer workstation. Three groups included a physician and a nurse, whereas the fourth included the pharmacist, admission officer, and clerk. The usability experts facilitated the walkthrough, tracked time, and gathered field observations.

We first gave each group written CLIPS and a patient summary. We then instructed groups to use the EHR to complete tasks, describe issues encountered, and assign each issue a usability criterion (i.e., “guidance,” “workload,” “compatibility,” “significance of codes,” “adaptability,” “error management,” “consistency,” or “explicit control”). Participants also assigned a severity level (i.e., “light,” “minor,” or “major”) to each issue. Please refer to Appendices B and C for criterion and severity definitions.^[29]^ We audio-recorded comments. The usability experts documented direct quotes during the sessions, issues reported by end-users, and criteria and severity scores. After each task, end-users completed a 4-item questionnaire adapted from Schumacher et al. with 5-point Likert-type items evaluating EHR features availability, completeness, ease of use, and efficiency.^[34]^ We published our questionnaire findings in a companion article.^[37]^

The walkthrough ended once participants completed all CLIPS or after two hours had elapsed. Afterward, users completed the System Usability Scale (SUS)^[38]^—a validated 10-item questionnaire with Likert-type statements and performance benchmarks. We published the results of this questionnaire in a companion article.^[37]^ We organized end-users into groups according to professional roles. A usability expert then debriefed each group using a semi-structured interview script exploring EHR strengths and weaknesses (Appendix D).

### Data Collection and Analysis

#### Question 1: Can end-users Detect, Describe, and Prioritize Usability Problems?

Our usability experts first read problems identified by the participants and excluded (1) those unrelated to specific EHR characteristics (e.g., opinions without descriptions), (2) those concerned with the technology platform (e.g., connection failures), or (3) those rooted in data upload problems. They then combined multiple descriptions of the same problem (i.e., deduplication) to reach a final list of usability problems.

Next, we created a usability expert “comparison set”. We combined lists of problems identified by participants and problems detected by usability experts. For each problem, we assigned a usability criterion and a severity level. Experts independently categorized problems using our *a priori* usability criteria and severity levels.^[29]^ Disagreements were resolved through consensus.^[28]^ We then compared end-users’ lists and assignments to the “comparison set.” We calculated concordance between end-users and experts using percent agreement and Krippendorf’s α.^[39]^ We also calculated the average issue detection rate within user profiles when there were multiple representatives (i.e., nurses and physicians).

#### Question 2: Does the Usability Walkthrough Method Identify Problems that Require Clinical Domain Expertise to be Detected?

Two usability experts screened problem descriptions to identify those requiring clinical expertise to detect. Since these represented new types of problems only clinicians recognized, our usability experts categorized each problem inductively.

#### Question 3: How Satisfied are end-users that Participate in a Usability Walkthrough?

After the last walkthrough, we asked participants to provide feedback on the method. To measure user satisfaction, we developed an eight-item questionnaire with 5-point Likert scales anchored by strongly disagree and strongly agree (Table 5). Participants also answered open-ended questions about the strengths and weaknesses of the method. We compared each rating to 3 (i.e., the median value) using the Wilcoxon sample signed-rank test with a significance threshold of 0.05. Two usability experts analyzed the qualitative data inductively to identify important or recurrent themes.

#### Question 4: What are the Challenges Associated with Implementing a Usability Walkthrough during an EHR Procurement Process?

We recorded all deviations from the workshop agenda and evaluation protocol. Two usability experts categorized each deviation according to the root cause.

## RESULTS

In this section, we report on the feasibility and utility of our usability evaluation strategy. We reported EHR usability findings in a companion article.^[37]^ Two vendors withdrew their applications during the procurement process, leaving only three candidate EHRs for analysis.

### Are End-Users Able to Detect, Describe, and Prioritize Usability Problems?

Participants reported 361 usability problems. We excluded 21 issues (5.82%) using our eligibility criteria. After deduplication, the final list consisted of 265 problems: 258 detected by end-users (97.36%) and 7 detected only by usability experts (2.64%) (Table 2). Each end-user within a professional role detected between 26.82 and 70.37% of all problems identified by that group (mean = 42.92%; SD = 14.11). On average, 59.83% of the problems were detected by one participant, 23.73% by two, and 12.75% by three.

**Table 2:** Frequency of Problems Detected per User Profile, and Number of End-Users Identifying the Problem (“0” Indicates Only the Usability Expert Identified a Problem)

User profile	Number of end-users detecting the problem	Number of problems detected (% per profile where relevant)			Sum
		EHR1	EHR 2	EHR3	
Physician	0 *(UE)*	1 (2.3%)	1 (2.4%)	1 (6.25%)	3
	1	31 (72%)	25 (61%)	8 (50%)	64
	2	8 (18.6%)	11 (26.8%)	4 (25%)	23
	3	3 (7%)	4 (9.7%)	3 (18.75%)	10
Nurse	0 *(UE)*	1 (3.8%)	0	2 (6.9%)	3
	1	15 (53.8%)	14 (63.6%)	17 (58.6%)	46
	2	8 (30.8%)	3 (13.6%)	8 (27.6%)	19
	3	3 (11.5)	5 (22.7%)	2 (6.9%)	10
Pharmacist	0 *(UE)*	0	0	0	0
	1	14 (100%)	23 (100%)	9 (100%)	46
Medical clerk	0 *(UE)*	0	0	0	0
	1	10 (100%)	9 (100%)	6 (100%)	25
Admission officer	0 *(UE)*	1 (20%)	0	0	1
	1	4 (80%)	5(100%)	6 (100%)	15
Sum		99	100	66	265

Acronyms: *UE*, usability expert; *EHR*, electronic health record

End-users assigned a criterion to 218 of the 258 problems (84.49%); 157 matched those assigned by usability experts (72%; Krippendorff’s *α* = 0.66 [0.59; 0.74], Table 3). The largest discrepancies were in three categories: “guidance,” “workload,” and “compatibility.” Participants assigned a severity level to 217 of the 258 problems identified (84.10%): 165 matched those assigned by experts (76%; Krippendorff’s α = 0.75 [0.68; 0.82], Table 4). End-users more often rated problems as “light” or "major,” whereas usability experts more often rated problems as “minor.”

**Table 3:** Number and Concordance of Usability Problems Per Criterion According to End-Users and Usability Experts. The Diagonal (Boldface) Indicates Agreement between Raters. This Table Does Not Include Emergent Issues and Categories Requiring Clinical Expertise to Identify (see Appendix E for This Data)

		End-users									
		Guidance	Workload	Compatibility	Signif. of codes	Error manag.	Consistency	Adaptability	Explicit control	No criterion	Sum
Usability experts	Guidance	**45**	7	4	3	2	1	3	1	16	82
	Workload	8	**32**	5	1	2	0	4	0	12	64
	Compatibility	1	4	**25**	1	0	1	5	0	6	43
	Significance of codes	0	1	1	**23**	0	0	0	0	2	27
	Error manag.	0	1	1	0	**15**	0	0	0	3	20
	Consistency	0	0	0	0	0	**11**	0	0	0	11
	Adaptability	1	0	0	1	0	0	**3**	0	1	6
	Explicit control	0	0	1	0	0	0	0	**3**	1	5
Sum		55	45	37	29	19	13	15	4	41	258

**Table 4:** Number of Usability Problems Per Severity Level Assigned by End-Users and Usability Experts. The Diagonal (Boldface) Represents Agreement in Severity Score between End-Users and Experts

		End-users				
		Light	Minor	Major	No severity	Sum
Usability experts	Light	**68**	6	0	21	95
	Minor	28	**63**	16	14	121
	Major	0	2	**34**	6	42
Sum		96	71	50	41	258

### Does the Usability Walkthrough Method Identify Problems That Require Clinical Domain Expertise to Be Detected?

Thirty-two of the 258 problems (12%) required clinical expertise to detect (Appendix E). In one instance, a pharmacist using the medication review module could not determine how to make a drug substitution, and in another, a physician did not receive an error message after entering the same treatment order twice. We include a complete list of issues in Appendix E. We classified these problems into 5 novel categories: patient identifiers (*n*=4), information availability and visibility (*n*=12), EHR configurations (*n*=9), access to EHR functions by users’ roles (*n*=2), and work and cognitive load problems (*n=*5).

There were instances when patient identifiers were not visible while performing tasks, increasing the risk of wrong patient selection errors. End-users encountered screens missing critical information, including orders, decision support, care plan actions, and medication changes. There were readability issues associated with typography and inappropriate “hard stops” (e.g., health insurance information queries blocking access to other functions). We found a mismatch between user EHR permissions and real-world scope of practice (e.g., nurses were granted access to prescribe when not permitted in clinical practice). Finally, the EHRs tended to increase workload and cognitive load. For example, some features increased the number of actions per task, failed to provide the user with feedback, or made selections confusing (e.g., a mismatch between medication type and dosing units).

#### How Satisfied are End-Users with the Usability Walkthrough?

Overall, end-users valued the usability walkthrough. The average score for each questionnaire item was at least four on a five-point scale (Table 5). Participants, however, struggled to assign usability criteria (*n*= 4 of 9), and all reported some challenges learning the scoring and categorization system. One respondent said, “*understanding the criteria takes time,*” and another would have appreciated “*even more training upstream of the evaluations*.” Nevertheless, most respondents (*n*= 8 of 9) said the method was “*easy to learn*.” Most participants liked the ability to quantify subjective impressions of the technology and indicated they would be willing to use this method again (*n*=8 of 9). They all agreed that the method “*clearly distinguished [EHRs] strengths and weaknesses,*” and the data permitted a “*detailed comparison of EHRs*.”

**Table 5:** Walkthrough Satisfaction Questionnaire Results and Statistical Comparison to the Median of 3 Using Wilcoxon Signed-Rank Test (scale: 1= Strongly Disagree; 5 = Strongly Agree)

Likert items	*N*	Mean	SD	Median	Range	W	*p*
I think I understood the usability walkthrough method	9	4.11	0.60	4	3 – 5	36	0.005
I felt efficient during the usability walkthrough	9	4.11	0.60	4	3 – 5	36	0.005
Using the usability walkthrough was easy for me	9	4	0.73	4	3 – 5	28	0.009
I think I understood the usability criteria	9	4.22	0.67	4	3 – 5	36	0.006
I feel able to evaluate the EHR software using the criteria	9	4	0.71	4	3 – 5	28	0.009
Using the usability criteria was easy for me	9	4	0.71	4	3 – 5	28	0.009
I found this method relevant for the EHR procurement process	8	4.38	0.52	4	4 – 5	36	0.006
I would appreciate to apply this method in other projects.	8	4.38	0.71	4	4 – 5	28	0.010

N = Number of respondents; SD = standard deviation, W = Wilcoxon test statistic; p = p-value

#### What Challenges Exist when Implementing a Usability Walkthrough during an EHR Procurement Process?

Two vendors withdrew their applications. One had concerns about their product’s usability; the other did not disclose a reason. The remaining vendors were not prepared for the evaluation. We could not complete all CLIPS due to technical issues (e.g., features not working) and database “locks” that prevented multiple users from opening the same test record simultaneously (Table 6). In some cases, the EHRs were missing patient data, whereas, in others, the data that participants were expected to enter were already in the chart.

**Table 6:** Number (and Percentage) of Tasks Completed by Each User Group for Each EHR

EHR	Nurse physician (group 1)	Nurse physician (group 2)	Nurse physician (group 3)	Medical secretary	Admission officer	Pharmacist
1	23/50 (46%)	31/50 (62%)	27/50 (54%)	3/3 (100%)	3/4 (75%)	2/2 (100%)
2	23/50 (46%)	28/50 (56%)	26/50 (52%)	3/3 (100%)	3/4 (75%)	2/2 (100%)
3	33/50 (66%)	42/50 (84%)	25/50 (50%)	3/3 (100%)	4/4 (100%)	2/2 (100%)

## DISCUSSION

### Principal Findings

Initial selection or transition to a new EHR can have seismic consequences on health system outcomes, patient safety, and clinician well-being.^[40]^ Choosing one that does not meet organizational needs can destabilize the work system and undermine patient safety. To our knowledge, this is the first study to evaluate the feasibility of a usability walkthrough involving end-users in EHR procurement. After a short training session, clinicians could identify, and risk stratify real EHR usability problems. The procurement team used our results to guide selection decisions and screen candidate EHRs for the third phase of the evaluation.

Our findings match studies of usability testing showing that testing products with as few as 3–5 users can identify up to 85% of interface problems affecting similar users.^[41]^ In our study, end-users within the same profile did not detect all the same problems. It is, therefore, crucial to recruit at least 3–5 evaluators per profile.^[42]^ While the assignment of criteria and scores by end-users and usability experts were consistent, end-users sometimes struggled to disambiguate “guidance,” “workload,” and “compatibility” criteria.^[43]^ Clinicians also tended to assign higher severity scores compared to usability experts. We hypothesize that the consensus method our experts used when assigning severity levels had a moderating effect on scores. Overall, our findings suggest that the usability walkthrough method—and the inclusion of end-users—is valid for identifying usability issues, is generalizable across settings, and may be extensible to other technologies.

End-users identified 32 usability problems that required clinical expertise to detect. This is consistent with studies showing that end-users with domain expertise identify problems that usability specialists may overlook.^[44]^ Some of the problems identified by clinicians could have had severe patient safety consequences. Patient identifier issues are known to increase the risk of wrong-patient prescribing^[45]^, missing patient data can affect medical decision-making, and inappropriate “hard stops” can delay patient care.^[8,46]^ Therefore, our findings reinforce the axiom that end-users must be engaged throughout the HIT lifecycle—including the procurement process.

### Feedback on Methods and Implications for Practice

Participants said they would use the walkthrough and scoring system for similar projects despite the learning curve. The information helped them objectively evaluate features and compare products systematically and argue preferences and advocate for their constituency. Data collected without vendor interference may generate more reliable information and more safety concerns than traditional product demonstrations, enabling business leaders to base decisions on technology elements or objective measures of human-computer interaction.^[31]^

### Lessons Learned and Limitations

While usability walkthrough methods do not require extensive training, there are opportunities to improve orientation and testing activities.^[47]^ Sending the CLIPS and sample patient data to the vendors could have given them an unfair advantage and influenced how they configured their EHRs. In practice, however, the vendors were unprepared for testing. We could not complete several test scripts because vendors did not include the minimum data required for each simulation. This may have biased our selection decisions. Before implementing the usability walkthrough, usability experts should complete a “cross-check” of the EHR setup. We hypothesize that France’s lack of incentives for usability work and evaluation could explain vendors’ lack of awareness surrounding prerequisites for usability evaluation (e.g., missing mock patient data in a test patient record). Usability requirements included in the ONC certification program and other federal research funding programs may have incentivized vendors to collaborate more effectively with US healthcare organizations and create better test environments.^[48]^

Readers should interpret the findings from our case study with caution. Our results are based on the behaviors and observations of a small sample of users recruited at a French hospital during an EHR procurement. The findings could differ across users, settings, products, or time points. Nevertheless, this case study shows how to gather usability data and stakeholder sentiments at the pace of healthcare operations.

Usability research adds up-front costs for the customer.^[49]^ The main costs include time spent creating test scenarios, recruiting participants, conducting usability evaluation, and retaining usability experts to proctor sessions, facilitate debriefs, and analyze the results.^[50]^ Executives must be convinced that the method generates a return on investment if they are to budget evaluation resources during procurement. The cost of testing must be weighed against the downstream costs of a poorly designed EHR related to training, workflow, efficiency losses, clinician burnout, staff disenfranchisement, patient endangerment, and legal actions.^[19]^

## CONCLUSIONS

The usability walkthrough is a feasible method to engage end-users in usability evaluation, compare EHRs during a procurement process, and galvanize buy-in for change among stakeholders. Nevertheless, efforts are needed to raise usability awareness and incentivize vendors to optimize product performance. Without strong federal policies or technical regulations, the procurement process represents an important lever for change.

### Supplementary Information

Below is the link to the supplementary material

Supplementary file1 (DOCX 33 KB)
