# Supplementary material for: Active Involvement of End-Users in an EHR Procurement Process: a Usability Walkthrough Feasibility Case Study
Source: J Gen Intern Med. 2023 Oct 5;38(Suppl 4):974–81. doi: 10.1007/s11606-023-08277-2 (PMC10593645; doi:10.1007/s11606-023-08277-2)
Supplement: Supplementary file 1 — Supplementary file1 (DOCX 33 KB) [file 11606_2023_8277_MOESM1_ESM.docx]

**Appendix 1.** Examples of clinical information processing scenarios (CLIPS)

| **CLIPS: Patient arrival at the Emergency Department** | | |
| --- | --- | --- |
| **Profile** | **Task** | **Patient information to enter / actions to take** |
| Admission officer | Create a record for a patient arriving in the emergency room | Mrs. Pabien Eva, wife Vamieux,  75 years old, born on 10/09/1945 in Roubaix  Address: 45 bis rue du bonheur, appt 45, résidence du Soleil, 59160 Lomme.  Health insurance number: 2 450959XXX XXX XX, complementary health insurance: Vespilly,  Attending physician Dr. Devallly Thimothé, e-mail address tdevallly@gmail.com, 067789XXXX,  Mode of entry: ambulance from home, non-medical transport |
| Nurse | Triage and orientation of the patient | Reason for admission: chest pain  Vital signs: Heart Rate 95, Saturation 97, Blood Pressure 11/8, Respiratory Rate 19, Pain Scale 7 out of 10, weight 70kg 1m65  Prioritization: 2  Administration of acetaminophen according to protocol  Orientation in the ward area: recumbent patients’ area |
| Physician | Medical observation | Reason for consultation: chest pain  History: diabetes, high blood pressure, atrial fibrillation  Treatment: metformin 1/d; amlodipine 5 1/d; amiodarone 200 5/d/7  Allergy: penicillin  Chest pain for 3 days with fever since that day  Cardiac auscultation: diastolic murmur not known  Pulmonary auscultation: clear  Neuro: clear  Hyperthermia  Conclusion: Suspicion of endocarditis, hospitalization in geriatrics |
| Physician | Ordering | Blood culture if temperature > 38°C |
| Physician | Medical decision | Endocarditis diagnosis / Clinical Classification of Emergency Patients |
| Nurse | Patient transfer to medical services | Transfer to geriatric service |

**Appendix 2.** Scapin and Bastien’s usability criteria

| Usability criterion | Definition |
| --- | --- |
| Guidance | Refers to the means available to advise, orient, inform, instruct, and guide the users throughout their interactions with a computer (messages, alarms, labels, etc.), including from a lexical point of view |
| Workload | Concerns all interface elements that play a role in the reduction of the users’ perceptual or cognitive load, and in the increase of the dialogue efficiency |
| Explicit control | Refers to the system processing of explicit user actions, and to the control users have on the processing of their actions by the system |
| Adaptability | Refers to system’s capacity to behave contextually and according to the users’ needs and preferences |
| Error management | Refers to the means available to prevent or reduce errors and to recover from them when they occur. Errors are defined in this context as invalid data entry, invalid format for data entry, incorrect command syntax, etc. |
| Consistency | Refers to the way interface design choices (codes, naming, formats, procedures, etc.) are maintained in similar contexts, and are different when applied to different contexts |
| Significance of  codes | Qualifies the relationship between a term and/or a sign and its reference. Codes and names are significant to the users when there is a strong semantic relationship between such codes and the items or actions they refer to |
| Compatibility | Refers to the match between users’ characteristics (memory, perceptions, customs, skills, age, expectations, etc.) and task characteristics on the one hand, and the organization of the output, input, and dialogue for a given application, on the other hand |

**Appendix 3.** Usability issue severity levels and descriptions

| Severity level | Definition |
| --- | --- |
| Light | At worst, the problem causes mild and easily overcome inconvenience |
| Moderate | The problem slows down or hinders task performance |
| Severe | The problem prevents task performance and may cause the user to make mistakes |

**Appendix 4.** Semi-structured interview script for the post-session debrief

For the difficulties/hesitations encountered but not commented by the participants but noted by the usability experts: we noticed that you had difficulties to perform [name of the task]. Can you tell me what were the causes?

[Once all difficulties are commented on]

What is your overall opinion of this EHR?

What do you think are the main benefits of this EHR? Why?

What are the main disadvantages of this EHR? Why?

How do you see yourself working with this EHR?

**Appendix 5.** Usability problems that required clinical expertise to detect, organized according to professional role. The EHR with the issue is denoted at the end of the text

|  | **Physicians** | **Nurses** | **Pharmacists** | **Medical Secretary** |
| --- | --- | --- | --- | --- |
| **Patient identifiers (*n*=4)** | Patient names are not visible enough on all pages of the patient record. After a disconnection, the displayed patient record has changed without the physician noticing: he has entered information on the wrong record. (EHR1) |  |  | On the appointment booking module the medical secretary uses a left arrow to go back. However, this arrow changes the patient record, and the secretary does not notice that the opened patient record has changed. (EHR1) |
|  | drug alerts for one patient appear while the physician is on another patient’s record (EHR3) |  |  | the patient's name does not appear on the screen, she does not know which patient she is on. (EHR2) |
| **Information availability and visibility (*n*=12)** | Not all the information entered is transmitted: e.g., out of 3 ICD10 codes entered in the emergency room, only 1 appears later in the patient record. (EHR3) | On the care plan, the samples to be taken are not visible, which means that there is a risk of not seeing them and not taking them (EHR3). | The pharmacist does not notice very small notifications when there is a change in a prescription, which prevents them from identifying prescriptions that need to be analyzed (EHR1) |  |
|  | The typography of the prescriptions is misleading: 1D sachet is easily confused with 10 sachets. (EHR 1) | The care plan is not legible and too small, there is a strong risk that nurses will miss information. (EHR1) | In the medication review module, pharmacists cannot find out how to make a drug substitution. Instead, they only find out how to make a proposal without a dose. (EHR1) |  |
|  | Some prescriptions are not visible to the physician (e.g., blood culture prescription), which can cause re-prescribing and prescription duplication problems. (EHR 2) |  | The pharmacist cannot enter a proposal for pharmaceutical intervention (EHR2) |  |
|  | After prescribing a 1L infusion, a 500mL infusion appears in the prescriptions, the other 500mL are not visible immediately. (EHR2) |  | The pharmacist cannot substitute medications that are not in the hospital's therapy booklet. (EHR2) |  |
|  | no error message after double entry of the same treatment. (EHR2, EHR3) |  |  |  |
| **Adaptation to practice (*n*=9)** | Some options are missing in the lists: the obstetrical history is not in the list of proposed history preventing to complete the file for a female patient. (EHR1) | Units for some data entries are missing (e.g., patient size) (EHR2, EHR 1) |  | It is mandatory to enter the health insurance card information before the patient can enter the emergency department, which may result in a loss of chance. (EHR2) |
|  | The dosing cycle is not adaptable to clinical need (e.g., it is not possible to order ¼ ¼ ½ Lexomil) (EHR2) | For some samples, a schedule is clinically necessary, but the software does not allow it. (EHR3) |  |  |
|  | Some protocols are not specific enough (e.g., "only once" is confusing). (EHR2) |  |  |  |
|  | Blood culture is listed twice in the prescription list: one corresponds to a care, the other to a blood test. Depending on the choice, the information given to the nurse is very different. The doctor does not see the difference between these two options in the list. (EHR2) |  |  |  |
|  | Impossible to enter conditional prescriptions. (EHR3) |  |  |  |
| **Access to EHR functions by users’ roles (*n*=2)** |  | Nurses access the drug prescribing interface when they are not legally authorized to prescribe. (EHR1) |  |  |
|  |  | The entry of the patient's discharge is assigned to the nurse in the software, whereas it is the admission officer who is responsible for this task. (EHR2) |  |  |
| **Work and cognitive load (*n*=5)** | When prescribing a medication, the list of units is long and includes units that are not relevant to the selected medication. (EHR1) |  | During the medication review, pharmacists do not have the possibility to refuse a prescription line easily: they can easily accept or refuse the whole prescription. But it makes them perform several actions to question and make a proposal for a single prescription line which adds an additional workload. (EHR1) |  |
|  | After the prescription entry, the physician does not know if the prescription is sent and seen by the nurses. (EHR1) |  |  |  |
|  | Blood culture is listed twice in the prescription list: one corresponds to a care, the other to a blood test. Depending on the choice, the information given to the nurse is very different. The doctor does not see the difference between these two options in the list. (EHR2) |  |  |  |
|  | On the conclusion window, the "save" and "validate" buttons are semantically ambiguous and do not have the same consequences (possibility or not to change the entered conclusion). However, when the conclusion is written by an intern, it must be reread by a senior before being definitively validated. (EHR3) |  |  |  |
